# Long non‐coding RNAs define favourable biology in high‐risk non‐muscle‐invasive bladder cancer

**DOI:** 10.1002/bco2.70131

**Published:** 2025-12-19

**Authors:** Rachel Weng, Tran Anh Thu Phung, Robert Bell, Lars Dyrskjøt, Ewan A. Gibb

**Affiliations:** ^1^ Vancouver Prostate Centre, M. H. Mohseni Institute of Urologic Sciences Vancouver BC Canada; ^2^ Department of Clinical Medicine Aarhus University Aarhus Denmark; ^3^ Department of Urologic Sciences University of British Columbia Vancouver BC Canada

**Keywords:** biomarkers, gene expression profiling, high‐risk bladder cancer, non‐coding RNA, risk stratification

## Abstract

**Background:**

To evaluate whether long non‐coding RNA (lncRNA) expression patterns can improve molecular stratification and outcome prediction in high‐risk non‐muscle‐invasive bladder cancer (NMIBC).

**Methods:**

RNA sequencing data from high‐grade Ta (TaHG) and T1 (n = 212) tumours from the UROMOL consortium (Lindskrog et al., Nature Communications 2021) were analysed. Unsupervised consensus clustering based on lncRNA expression patterns identified distinct patient subgroups, which were characterized using gene expression patterns and gene signatures. A single‐sample classifier was trained using elastic net logistic regression on UROMOL lncRNA expression profiles and applied to the Knowles cohort for independent validation. Recurrence‐free survival (RFS) and progression‐free survival (PFS) were evaluated using Kaplan–Meier (KM) plots, univariate and multivariate analyses.

**Results:**

LncRNA expression patterns identified three distinct clusters of TaHG and T1 tumours (LC1, LC2, LC3). Of these, the LC1 subgroup (n = 47) had significantly better RFS (p = 0.04) and PFS (p = 0.002). The LC1 subgroup was characterized by downregulation of genes associated with proliferation (i.e., *FOXM1*, *MKI67*) and lower G2M and E2F gene signatures, suggesting reduced rates of tumour growth. A transcriptomic classifier trained on UROMOL lncRNA profiles successfully stratified recurrence risk in an independent validation cohort (Knowles, n = 120), where predicted high‐risk cases (LC2/3) demonstrated significantly poorer recurrence‐free survival (p < 0.001). While these findings highlight lncRNA expression as a potential stratification tool, limitations include the retrospective design, treatment heterogeneity and the need for external validation.

**Conclusion:**

LncRNA‐based clustering demonstrates significant potential for improving patient stratification in high‐risk NMIBC, identifying less aggressive tumours in an otherwise high‐risk setting. A transcriptomic classifier trained on these findings was successfully validated in an independent cohort, supporting its potential clinical utility in refining risk assessment and guiding treatment decisions. Prospective studies are needed to further validate and refine this approach.

## 1 BACKGROUND

High‐risk non‐muscle invasive bladder cancer (NMIBC), which includes both high‐grade stage Ta and T1 tumours, is a heterogeneous disease with variable clinical outcomes.^1^, ^2^ Patients with high‐risk NMIBC face a substantially elevated risk of both recurrence and progression, often necessitating early and aggressive treatment strategies.^2^, ^3^, ^4^ Standard‐of‐care for high‐risk disease involves transurethral resection of the bladder tumour (TURBT) followed by intravesical Bacillus Calmette‐Guérin (BCG) immunotherapy. Despite this approach, long‐term disease control is achieved in only approximately 40% of patients, spurring the investigation of numerous alternative therapeutic options in clinical trials.^5^, ^6^, ^7^, ^8^, ^9^ Accordingly, there remains a critical unmet need for biomarkers that can enhance patient stratification and more accurately identify which patients require escalated therapeutic interventions as well as those who could safely be de‐escalated.

Gene expression‐based molecular subtyping has been used to investigate tumour biology in muscle invasive bladder cancer (MIBC), where it has potential as a prognostic and predictive biomarker.^10^, ^11^, ^12^, ^13^, ^14^ Broadly, MIBC can be divided into luminal and non‐luminal, including basal‐squamous and neuroendocrine‐like, with later frameworks such as the Lund taxonomy providing additional granularity.^15^, ^16^, ^17^, ^18^ When these MIBC models are applied to NMIBC, however, nearly all tumours are classified as luminal‐papillary or UroA, which limits their clinical utility in this setting.^19^, ^20^, ^21^ Accordingly, NMIBC‐specific classification systems have been developed, particularly for high‐risk disease, with UROMOL 2021 providing the most extensively validated classifier, while other models remain less well tested in external cohorts.^22^

In high‐risk NMIBC, transcriptome‐based subtypes have also advanced understanding of tumour biology and clinical behaviour.^22^ The UROMOL 2021 study defined four classes across a large cohort of Ta and T1 tumours and linked them to distinct outcomes, with Class 2a associated with poorer RFS and PFS, and Class 1 with more favourable prognosis.^19^ Other groups have reported alternative three‐class or five‐class solutions for T1 disease, with distinct biology and associations with BCG response or recurrence (for example, BRS1–3; T1‐Myc, T1‐Luminal, T1‐Inflammatory, T1‐Early and T1‐MYC‐enriched) across cohorts of varying size.^20^, ^23^ Stage‐stratified approaches have further identified Ta‐focused expression groups (TaE1–E3) and T1‐focused clusters (T1E1–E4), with differences in recurrence risk and treatment outcomes.^24^ Collectively, this body of work has shown that transcriptional programs capture clinically relevant heterogeneity in high‐risk NMIBC, but variability in cohort size, analytic platforms and external validation has limited the translation of any single scheme into routine practice.

Messenger RNA (mRNA) expression profiles have been expanded to include long non‐coding RNA (lncRNA) profiles in efforts to identify biomarkers for bladder cancer.^11^, ^25^, ^26^, ^27^ LncRNAs are mRNA‐like transcripts that are at least 200 nucleotides long but lack functional open reading frames, and they may or may not be polyadenylated.^28^, ^29^ These transcripts often constitute a significant portion of the transcriptome, and while their biological roles are not yet fully understood, their expression is often highly tissue‐specific or disease‐specific, suggesting their strong potential as biomarkers.^30^, ^31^ In bladder cancer, lncRNA expression profiles have been shown to define clinically relevant subdivisions, identifying patients with prognostic potential in both MIBC and NMIBC or those with micropapillary‐like disease in luminal T1 NMIBC.^11^, ^16^, ^25^, ^32^, ^33^

In this study, we queried the UROMOL database of high‐risk NMIBC samples and identified a subgroup of high‐risk NMIBC patients with less aggressive biological profiles. These patients have lower rates of recurrence and progression events and may be candidates for treatment de‐escalation.

## 2 METHODS

### 2.1 Patient cohorts

Patient cohorts were obtained from the UROMOL consortium^19^ and the Knowles study,^24^ both of which included gene expression profiles and clinical annotations. For UROMOL, bulk RNA sequencing data and normalized expression profiles were previously described and are available in the supplementary materials of the 2021 publication. For Knowles, microarray data generation and processing were previously reported. In this study, we focused on high‐risk, high‐grade Ta (TaHG) and T1 tumours, analysing 212 cases from UROMOL and 120 cases from Knowles.

### 2.2 Gene expression profiling

Details regarding the generation of RNA‐seq profiles for the UROMOL cohort have been previously described,^19^ and normalized gene expression data were provided in the supplementary materials of that study. Microarray data for the Knowles cohort were similarly generated and published in prior work.^24^

### 2.3 Unsupervised consensus clustering

Using Ensembl 112 (May 2024), we identified 9139 lncRNA features that were available among the UROMOL gene expression data matrix. The lncRNA expression data were pre‐processed using median absolute deviation (MADS) to identify highly variant lncRNA features for unsupervised clustering analyses (R package ConsensusClusterPlus, ver. 1.70.0). Unsupervised consensus clustering was performed on gene sets ranging from 500 to 2000 gene features. Outputs from ConsensusClusterPlus (including tracking plots, delta plots and cumulative distribution function [CDF] plots) were evaluated, and a 1000‐gene feature solution was selected as most appropriate and informative. The expression clustering analysis was performed using the partitioning around medoids (PAM) algorithm and Pearson correlation distance, with Ward's minimum variance method (Ward.D2) used for hierarchical clustering. Clustering was performed for 500 iterations, resampling 95% of samples (pItem = 0.95) in each iteration.

### 2.4 Classification of tumours into molecular mRNA subtypes

To assign tumours to the mRNA‐based molecular bladder cancer subtypes, we downloaded R packages ‘consensusMIBC’ and ‘BLCAsubtyping’ from GitHub and applied the Consensus and TCGA subtyping models to the normalized expression data, respectively.^15^ For the LundTax subtypes, we downloaded and applied the package ‘LundTaxonomy2023Classifier’ from GitHub.^34^ The UROMOL2021 subtyping classes were available as part of the supplemental data from the corresponding manuscript.^19^

### 2.5 Gene expression analyses

Heatmaps and boxplots were used to visualize differences between de novo generated unsupervised lncRNA‐based consensus clusters (LCs), using cluster‐specific selected lncRNAs, genes and hallmark gene sets from the molecular signature database hallmark gene set collection (MSigDB).^35^ The sonic hedgehog (SHH) pathway and immune190 score calculations have been described previously.^11^ For all differential expression and pathway analyses, false discovery rate (FDR) correction was applied using the Benjamini‐Hochberg method to account for multiple testing.

### 2.6 Statistical analyses

All statistical analyses were performed using R statistical software (R Foundation for Statistical Computing, Vienna, Austria). Patient and tumour characteristics were compared between subgroups by using X^2^ tests and two‐sided Wilcoxon rank‐sum tests. *P* values in boxplot figures represent results of Kruskal‐Wallis rank sum tests when comparing multiple groups, and Wilcoxon rank sum tests when comparing two groups. The primary endpoints were recurrence‐free survival (RFS) and progression‐free survival (PFS); however, PFS was not evaluated in the Knowles cohort due to a lack of progression events. Patients who were lost to follow‐up were censored at the date of last contact. The Kaplan–Meier method was used to estimate survival probabilities, and statistical significance of differences between survival curves for patients of different molecular subgroups was assessed using the log‐rank test. Multivariable analyses were performed using Cox proportional hazards models, adjusting for clinical covariates.

### 2.7 Discovery and validation of a transcriptomic classifier

A single‐sample classifier was trained on lncRNA expression data from the UROMOL cohort using elastic net‐regularized logistic regression (α = 0.5). Patients were grouped into LC1 (favourable outcome) and LC2/3 (unfavourable outcome) subtypes based on unsupervised consensus clustering and recurrence‐free survival analysis. Feature selection was performed using a nested cross‐validation approach, identifying a robust set of 179 lncRNAs predictive of subgroup membership. A final elastic net model was trained on the full UROMOL cohort using this feature set, resulting in 116 lncRNAs with non‐zero coefficients. The trained classifier was then applied to the Knowles cohort to assign patients to LC1 or LC2/3 subgroups using an optimal classification threshold derived from the training data.

## 3 RESULTS

The clinicopathological characteristics of the UROMOL and Knowles cohorts are outlined in Table 1. The UROMOL cohort (n = 212) was predominantly male (80%) with a median age of 71 years. Most tumours were TaHG, and 65% of patients experienced recurrence, with 20% progressing to MIBC over a median follow‐up of 48.5 months. Intravesical treatment was common (57% received MMC, 37% BCG). The Knowles cohort (n = 120), used for validation, was similar in age and sex but had more T1HG tumours and greater use of BCG (52%), with limited MMC exposure and no tumour size data available.

**TABLE 1:** Clinical characteristics of the UROMOL and Knowles cohorts.

	UROMOL	Knowles
N	212	120
TaHG	111	33
T1HG	101	87
Median Age (IQR)	71 (64–79)	73 (66–78)
Male sex	169 (79.7%)	95 (79.2%)
CIS		
Yes	44	37
No	168	67
NA	0	16
MMC or other		
Yes	120 (56.6%)	6 (5.0%)
No	82 (38.7%)	114 (40.4%)
NA	10 (4.7%)	0 (0.0%)
BCG		
Yes	79 (37.3%)	62 (51.7%)
No	133 (62.7%)	58 (48.3%)
Tumour size		
< 3 cm	127 (59.9%)	0 (0.0%)
**≥**3 cm	49 (23.1%)	0 (0.0%)
NA	36 (17.0%)	120 (100.0%)
Purpose	Discovery & Training/Testing	Validation

IQR: interquartile range, MMC: Mitomycin C, BCG: Bacillus Calmette–Guérin.

### 3.1 Consensus clustering of high‐risk NMIBC identified three groups of patients with distinct long non‐coding RNA expression profiles

**FIGURE 1:**
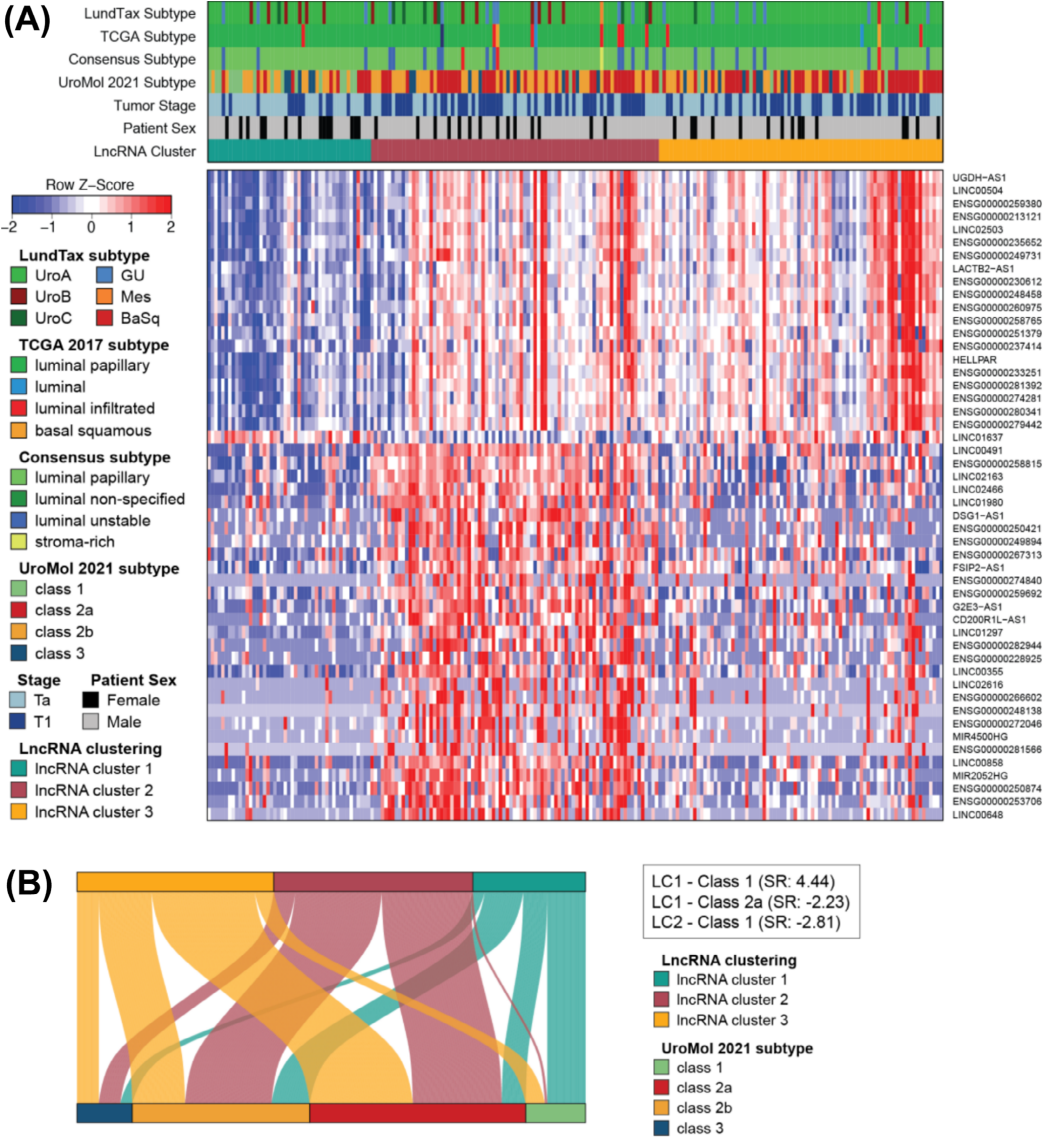
Characterization of a three‐cluster unsupervised solution derived from lncRNA expression profiles. (A) Heatmap of the top 50 differentially expressed lncRNAs defining the clustering solution. Covariate tracks indicate molecular subtypes according to LundTax, TCGA, consensus, and UROMOL classification systems, alongside tumour stage and patient sex. lncRNAs were selected based on adjusted p‐value and expression variance to highlight features driving the clustering. (B) Association between the lncRNA‐defined clusters and UROMOL subtypes. A Sankey plot illustrates subtype distributions across clusters, with significant standard residuals (SR) from the contingency analysis highlighted in boxes to the right.

Unsupervised consensus clustering of the top 1000 most variable lncRNAs revealed a robust three‐cluster solution (Figure S1), with strong internal coherence in two clusters and moderate structure in the third. To identify cluster‐enriched lncRNAs, we performed differential expression analysis comparing each cluster against the remainder of the cohort using stringent criteria (|log₂FC| > 1; FDR‐adjusted p < 0.05). This yielded 342, 164 and 55 significantly differentially expressed lncRNAs in clusters LC1, LC2 and LC3, respectively, for a total of 420 unique genes. The top 50 lncRNAs, ranked by minimum adjusted p‐value across all contrasts, were prioritized for visualization, revealing distinct expression patterns in LC1 and greater overlap between LC2 and LC3 (Figure 1A).

Cross‐comparison with established subtyping frameworks confirmed strong overlap with luminal phenotypes. The majority of tumours were classified as UroA (167/212; 78.8%) by the LundTax model, luminal papillary (198/212; 93.4%) by the TCGA 2017 model and LumP (184/212; 86.8%) by the Consensus classification system (Figure 1A).

In contrast, the UROMOL 2021 subtypes were more broadly distributed across the three lncRNA clusters (Figure 1A, Table S1). LC1 was enriched for Class 1 and 2b (16/47 each), LC2 was dominated by Class 2a (37/83) and 2b (36/83), while LC3 skewed toward Class 2a (43/82). Class 3 was generally underrepresented. A chi‐squared test confirmed a significant association between lncRNA clusters and UROMOL subtypes (χ^2^ = 38.1, df = 6, p < 0.001; Table S2). Although prior UROMOL sub‐analyses stratified Ta low‐grade and T1 high‐grade tumours also reported additional heterogeneity at the mRNA level, these refinements did not consistently improve outcome prediction.^19^ These relationships are further illustrated in a Sankey plot, with dominant transitions highlighted by standardized residuals ≥ ± 2, most notably LC1‐Class 1, LC1‐Class 2b and LC2‐Class 2a (Figure 1B, Table S3).

### 3.2 Biological characterization reveals a less aggressive expression profile in lncRNA cluster 1

As with the lncRNA analysis, we performed differential expression profiling of coding genes using identical thresholds (|log₂FC| > 1; FDR‐adjusted p < 0.05). This identified 564, 471 and 146 significantly differentially expressed genes in clusters LC1, LC2 and LC3, respectively, corresponding to 884 unique coding genes. Heatmap visualization of these genes revealed that LC1 was the most transcriptionally distinct, though it shared some weaker structural similarity with LC3. In contrast, LC2 and LC3 exhibited broadly similar gene expression patterns, yet remained clearly distinguishable (Figure S2).

The LC1 cluster was notably depleted in many genes, including several regulators of cell proliferation and mitotic spindle formation such as *MKI67*, *ASPM* and *CENPF* (Figure S2). In contrast, LC1 demonstrated elevated expression of genes associated with immune infiltration, including *CCL15* and *PSMB10*, both linked to T‐cell recruitment. Notably, *RNH1* was also upregulated; lower expression of this gene has been associated with increased invasion and metastasis in bladder cancer, suggesting a protective role in this context.

The LC2 cluster showed specific enrichment for an unusual panel of genes. These included *MAGEA3*, *MAGEA6* and *MAGEA10*, all cancer‐testis antigens previously associated with higher tumour grade, stage, and risk of invasion in NMIBC. LC2 also exhibited elevated expression of *NEB*, a gene more commonly linked to neuroendocrine and small cell bladder cancer, as well as *NELL2*, which has been implicated in promoting cell viability and proliferation in bladder cancer cells. In contrast, the LC3 cluster was less distinct, sharing expression patterns with both LC1 and LC2, but without the same degree of specificity or enrichment.

**FIGURE 2:**
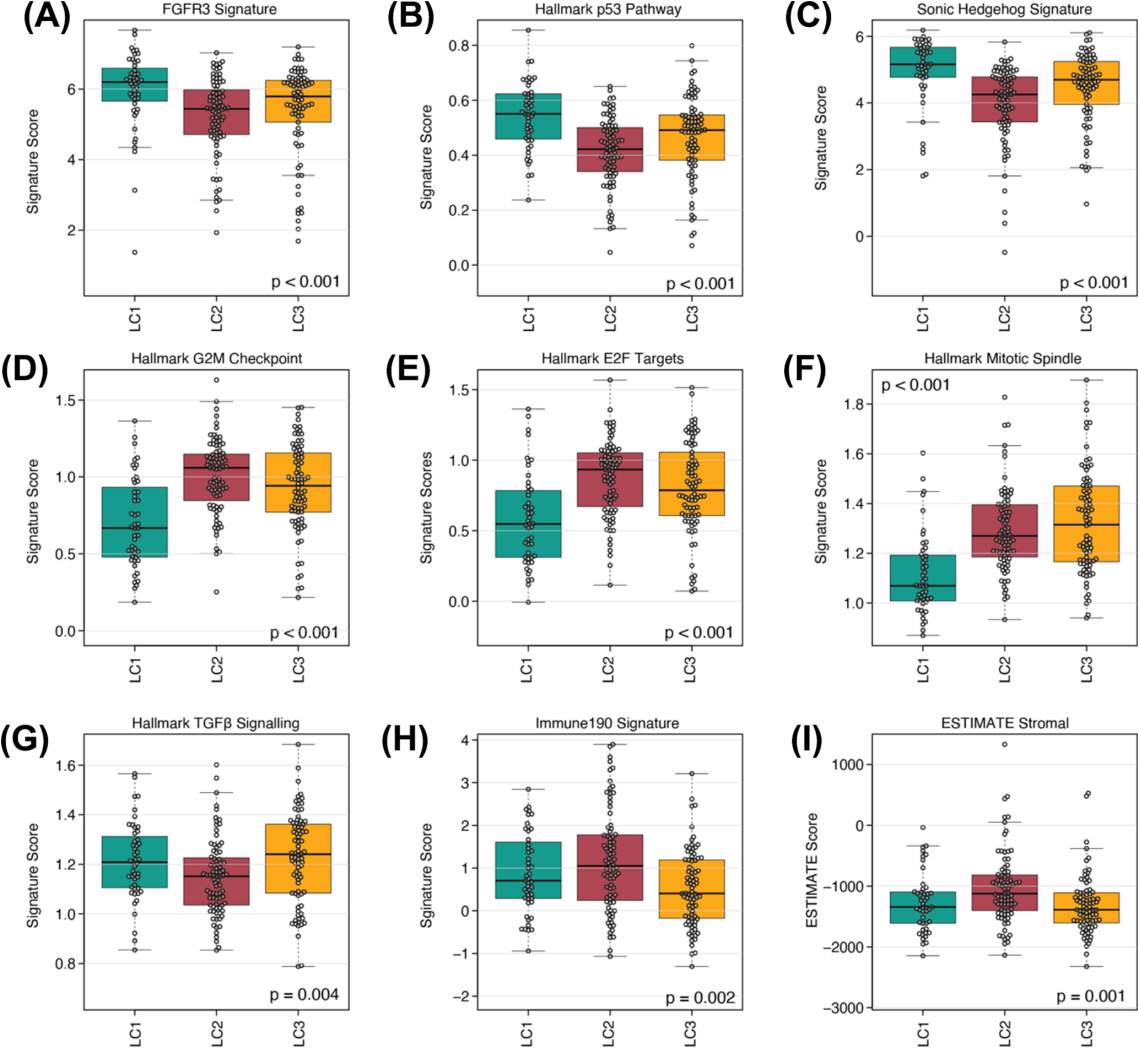
Further characterization of the lncRNA clusters using gene expression signatures. Each panel shows the distribution of signature scores across the three lncRNA‐defined clusters, highlighting differences in oncogenic signalling, proliferation, and tumour microenvironment composition. Signatures include: (A) FGFR3, (B) p53 activity, (C) sonic hedgehog (SHH), (D) G2M checkpoint, (E) E2F targets, (F) mitotic spindle, (G) TGFβ signalling, (H) immune190, and (I) ESTIMATE stromal score.

Next, we assessed enrichment of the MSigDB Hallmark collection along with selected immune‐ and stromal‐associated gene signatures. LC1 presented the highest activity scores for FGFR3, p53 and Sonic Hedgehog (SHH) pathways compared to the other clusters (Figure 2A–C). In contrast, hallmark signatures related to G2M checkpoint, E2F targets and mitotic progression were markedly lower in LC1, consistent with a non‐proliferative transcriptional profile (Figure 2D–F). By comparison, TGFβ signalling, Immune190 and ESTIMATE stromal scores showed only modest inter‐cluster differences (Figure 2G–I), indicating that the lncRNA clusters are not strongly distinguished by immune or stromal composition.

### 3.3 LncRNA cluster 1 is associated with reduced risk of disease recurrence and progression

**FIGURE 3:**
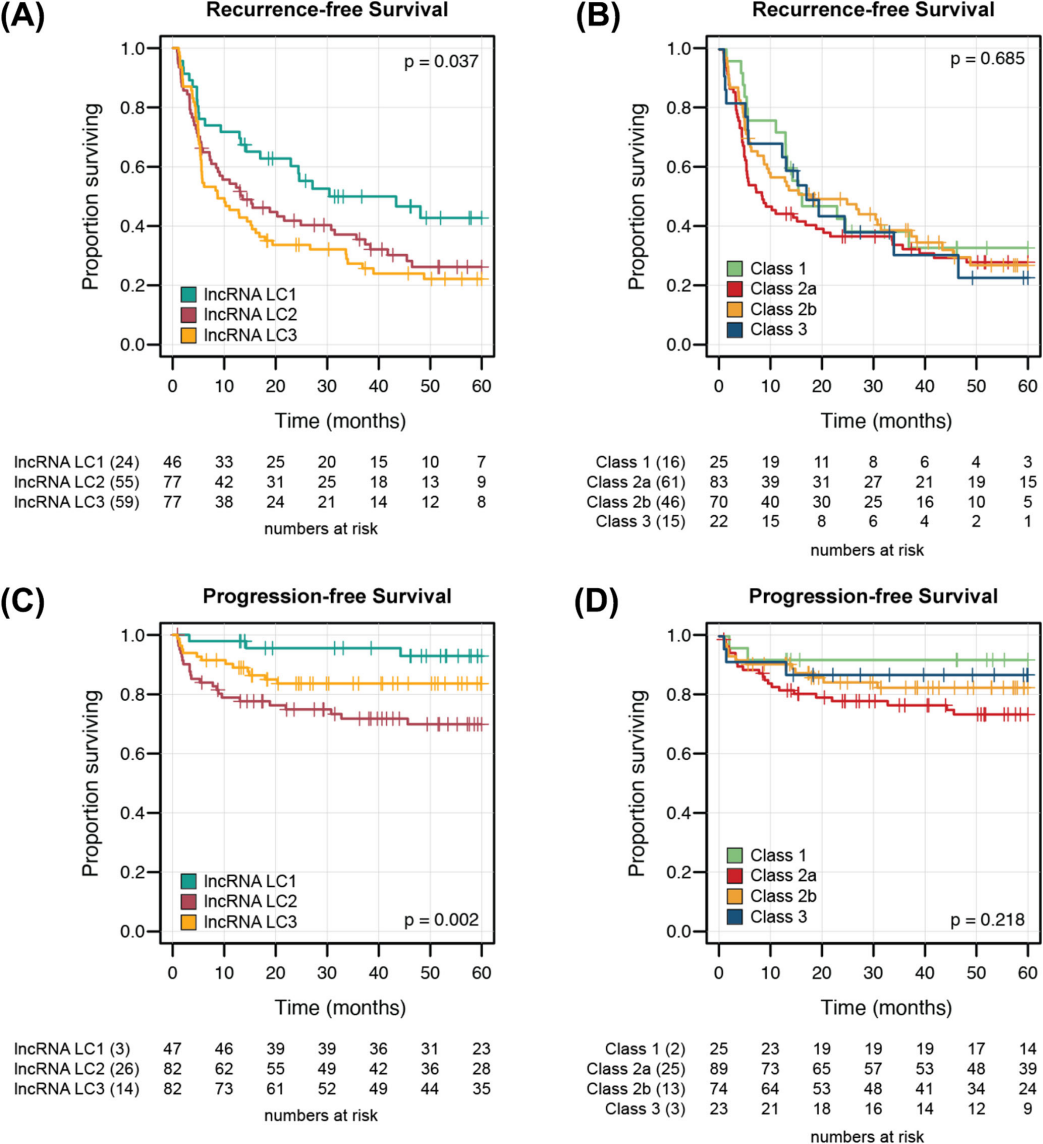
Clinical outcomes for 212 high‐grade Ta and T1 bladder cancer cases. Kaplan–Meier survival analyses showing recurrence‐free survival stratified by (A) lncRNA‐defined clusters and (B) UROMOL subtypes, and progression‐free survival stratified by (C) lncRNA‐defined clusters and (D) UROMOL subtypes. P‐values were calculated using the log‐rank test.

To evaluate whether our lncRNA‐based clusters were associated with distinct clinical behaviour, we assessed recurrence‐free survival (RFS) and progression‐free survival (PFS) using Kaplan–Meier analysis and compared these results to the UROMOL2021 subtypes (Figure 3).

For RFS, patients in LC1 demonstrated significantly improved outcomes compared to those in LC2 and LC3, which showed similar prognoses (p = 0.037; Figure 3A). In contrast, the UROMOL2021 subtypes did not significantly stratify patients by recurrence risk (p = 0.685; Figure 3B). For PFS, our clustering solution identified three groups with distinct prognosis: LC1 again showed the most favourable outcomes, while LC3 was associated with the poorest prognosis (p = 0.002; Figure 3C). Although the UROMOL2021 subtypes showed a similar pattern, with Class 1 tumours having the best outcomes and Class 2a the worst, this separation did not reach statistical significance (p = 0.218; Figure 3D).

Multivariable Cox proportional hazards models controlling for age, sex, tumour stage, BCG treatment and presence of concomitant CIS confirmed the prognostic relevance of LC1 for both RFS and PFS (Tables 2, 3). In the RFS model, LC1 was independently associated with reduced recurrence risk (HR 0.58, 95% CI 0.37–0.90, p = 0.02; Table 2). Concomitant CIS was also significantly associated with increased risk in this model (HR 1.96, 95% CI 1.26–3.1, p = 0.003). For PFS, tumour stage (Ta) (HR 0.20, 95% CI 0.10–0.43, p < 0.001), receipt of BCG (HR 0.23, 95% CI 0.10–0.52, p < 0.001) and LC1 membership (HR 0.58, 95% CI 0.37–0.90, p < 0.001) were each protective, while LC2 was associated with increased risk of progression (HR 2.39, 95% CI 1.26–4.50, p = 0.007) (Table 3).

**TABLE 2:** Univariable and multivariable cox proportional hazards analysis for time‐to‐recurrence in high‐risk NMIBC (n = 212). Variables include clinical covariates and lncRNA cluster membership (LC1–LC3) derived from the UROMOL cohort. Hazard ratios (HR) and 95% confidence intervals (CI) are shown.

N = 212	Univariable		Multivariable	
Recurrence	HR (CI)	P value	HR (CI)	P value
Age	1.003 (0.986–1.021)	0.734	1.004 (0.985–1.022)	0.700
Gender (M)	0.776 (0.515–1.170)	0.226	0.694 (0.452–1.067)	0.096
Tumour stage (Ta)	0.891 (0.637–1.247)	0.502	0.988 (0.690–1.413)	0.945
BCG ever in disease course	1.166 (0.827–1.642)	0.381	0.806 (0.528–1.232)	0.320
Concomitant CIS (Yes)	1.934 (1.322–2.830)	0.001	1.987 (1.279–3.087)	0.002
C1 (against others)	0.580 (0.373–0.903)	0.016	0.556 (0.340–0.908)	0.019
C2 (against others)	1.081 (0.768–1.522)	0.654		
C3 (against others)	1.360 (0.970–1.908)	0.074		

**TABLE 3:** Univariable and multivariable cox proportional hazards analysis for time‐to‐progression in high‐risk NMIBC (n = 212). Variables include clinical covariates and lncRNA cluster membership (LC1–LC3) derived from the UROMOL cohort. Hazard ratios (HR) and 95% confidence intervals (CI) are shown.

N = 212	Univariable		Multivariable	
Progression	HR (CI)	P value	HR (CI)	P value
Age	1.022 (0.991–1.055)	0.164	1.005 (0.975–1.037)	0.748
Gender (M)	1.125 (0.519–2.438)	0.765	0.759 (0.341–1.691)	0.500
Tumour stage (Ta)	0.234 (0.115–0.475)	0.000	0.199 (0.096–0.414)	0.000
BCG ever in disease course	0.380 (0.181–0.800)	0.011	0.238 (0.104–0.541)	0.001
Concomitant CIS (Yes)	0.924 (0.441–1.934)	0.833	1.026 (0.440–2.395)	0.952
C1 (against others)	0.226 (0.070–0.735)	0.013	0.244 (0.073–0.818)	0.022
C2 (against others)	2.623 (1.421–4.840)	0.002		
C3 (against others)	0.770 (0.405–1.462)	0.424		

### 3.4 Discovery and validation of a single‐sample transcriptomic classifier

To assess whether our lncRNA‐based classification captured prognostic differences beyond the UROMOL cohort, we trained a single‐sample transcriptomic classifier (TC) and applied it to high‐risk NMIBC tumours from an independent dataset (Knowles, n = 120).

The 25 of 120 tumours predicted as LC2/3 by the classifier showed biological profiles consistent with the original UROMOL clusters, including significantly lower FGFR3 (p = 0.045), p53 (p = 0.030) and SHH (p = 0.045) pathway activity (Figure S3). These tumours also demonstrated higher median proliferation signature scores, although this difference did not reach statistical significance (Figure S3).

Importantly, despite the overall lower recurrence risk in the Knowles cohort, the classifier significantly stratified recurrence‐free survival (p < 0.001; Figure S4A). After adjusting for tumour stage, BCG treatment, CIS, age and sex in multivariable analysis, predicted LC2/3 status remained independently associated with increased recurrence risk (HR = 2.50, 95% CI: 1.36–4.62, p = 0.003; Figure S4B)

## 4 DISCUSSION

High‐risk NMIBC (i.e., TaHG, T1) is marked by frequent recurrence, with T1 disease carrying a significantly greater risk of progression.^36^ These aggressive clinical presentations prompt intensified treatment, such as intravesical BCG or radical cystectomy. However, treatment escalation risks overtreating patients whose disease may not warrant such interventions. Accurately identifying patients at highest risk of recurrence or progression remains a major clinical challenge, highlighting the need for robust biomarkers to guide risk stratification and personalized management. In this study, we used long non‐coding RNA (lncRNA) expression patterns and unsupervised consensus clustering to stratify high‐risk NMIBC into subgroups with distinct prognoses.

Historically, lncRNA expression signatures have demonstrated utility across a spectrum of bladder cancer contexts. In luminal muscle‐invasive bladder cancer (MIBC), lncRNA profiling identified a subset of tumours with more favourable outcomes and enriched FGFR3 signalling, suggesting potential candidates for FGFR3‐targeted therapy.^11^, ^37^ We and others have extended these insights to non‐muscle‐invasive disease, where lncRNA profiles revealed micropapillary‐like T1 tumours with poor progression‐free survival^25^ and defined molecular distinctions within the UROMOL NMIBC cohort.^33^ More recently, we showed that lncRNAs could uncover aggressive biology even in clinically low‐risk, low‐grade Ta tumours,^32^ underscoring the specificity and broad applicability of lncRNAs as molecular classifiers across bladder cancer subtypes.

Most tumours in both cohorts were classified as luminal by the TCGA and Consensus classifiers, and the LundTax model offered slightly greater granularity. However, these molecular subtyping models did not provide additional prognostic value beyond standard clinical factors such as stage. This contrasts with MIBC, where luminal tumours typically have better outcomes^11^, ^16^, ^37^ and underscores the need for additional approaches to resolve clinically meaningful risk groups within high‐risk NMIBC. Our lncRNA clustering solution provided this refinement, identifying biologically and clinically distinct subgroups. LC1, which had the most favourable recurrence and progression outcomes, predominantly aligned with UROMOL Class 1 (the least aggressive subtype) but also captured cases from other classes. In contrast, LC2 and LC3 were composed primarily of tumours classified as Classes 2a, 2b and 3, subtypes generally associated with higher risk.

The lncRNA‐defined clusters reflected distinct biological programs that aligned with clinical outcomes. LC1 tumours, which had the most favourable prognosis, displayed a differentiated luminal profile characterized by active FGFR3, p53 and Sonic Hedgehog (SHH) signalling features associated with less aggressive disease in MIBC.^2^, ^7^ Reduced activity of proliferative pathways (G2M checkpoint, E2F targets, mitotic spindle) and lower expression of cell cycle regulators such as *MKI67*, *ASPM* and *CENPF* further suggested a less proliferative phenotype. LC1 tumours also showed modest enrichment for immune‐associated transcripts, including *CCL15* and *PSMB10*, and elevated *RNH1* expression, a gene previously linked to reduced invasion and metastasis in bladder cancer. Together, these findings support the idea that favourable FGFR3‐driven luminal biology, coupled with reduced proliferation, defines a low‐risk subset within high‐grade NMIBC.

In contrast, LC2 tumours harboured a transcriptional profile associated with more aggressive behaviour. This included strong upregulation of cancer‐testis antigens (*MAGEA3*, *MAGEA6*, *MAGEA10*) and neuroendocrine‐associated genes such as *NEB* and *NELL2*, features linked to higher tumour grade, invasion and lineage plasticity in bladder cancer. LC3 lacked a dominant biological program, sharing elements of both LC1 and LC2, and may represent a transcriptionally heterogeneous or intermediate group. Clinically, these differences aligned with outcomes: LC1 tumours had the most favourable trajectories, while LC2 was associated with the worst progression, reinforcing the biological distinction between clusters. Importantly, a classifier trained on UROMOL lncRNA profiles predicted recurrence risk in an independent, less aggressive cohort (Knowles), underscoring the potential utility of transcriptomic stratification in guiding surveillance intensity or adjuvant therapy decisions.

This study has several limitations. First, the retrospective design may introduce bias and limits generalizability. Second, the size of the cohorts restricted subgroup analyses, such as separating TaHG from T1 tumours. Third, differences in the transcriptomic platforms between the training (UROMOL, RNA‐seq) and validation (Knowles, microarray) cohorts may affect classifier reproducibility and performance. In addition, the low number of progression events in Knowles limited external validation to recurrence‐free survival. Finally, while the classifier demonstrated prognostic value, it remains preliminary and requires further refinement and prospective validation.

## AUTHOR CONTRIBUTIONS

RW conducted the bioinformatics analyses and figure generation. TATP developed the classifier model. RB supported the bioinformatics analysis and manuscript revision. LD provided cohort data, contributed to data interpretation and revised the manuscript. EAG supervised the study, drafted and critically revised the manuscript and served as guarantor of the work. All authors read and approved the final manuscript.

## CONFLICT OF INTEREST STATEMENT

The authors declare that they have no competing interests.

## ETHICS APPROVAL AND CONSENT TO PARTICIPATE

This study involved secondary analysis of de‐identified, publicly available transcriptomic datasets (UROMOL2021 and Knowles et al. 2021). According to local regulations, no new patient recruitment or direct patient involvement occurred. Ethics approval and consent to participate were therefore not required.

## CONSENT FOR PUBLICATION

Not applicable. This manuscript does not contain any individual person's data in any form.

## Supporting information


**Table S1:** UROMOL 2021 classes for each of the four lncRNA clusters.
**Table S2:** Expected frequencies for the Chi‐squared test.
**Table S3:** Standard residuals for the X2 test comparing the lncRNA clusters with UROMOL 2021 classes.

**Figure S1:** Consensus clustering of lncRNA expression in high‐risk NMIBC. Consensus matrix heatmap of 212 UROMOL tumours using the top 1000 variable lncRNAs. A three‐cluster solution (C1–C3) was identified, with dark blue blocks indicating stable membership across resampling iterations.
**Figure S2**. Biological characterization of the lncRNA‐based clusters. Heatmap of the top 50 most differentially expressed protein‐coding genes across the three lncRNA‐defined clusters. Covariate tracks indicate molecular subtypes according to LundTax, TCGA, Consensus and UROMOL classifications, as well as tumour stage and patient sex. Genes were selected based on adjusted p‐value and fold‐change criteria, and highlight biological programs distinguishing each cluster.
**Figure S3. Biological characterization of classifier‐predicted subgroups in the Knowles cohort.** Boxplots showing the distribution of signature scores across predicted LC1 and LC2/3 groups in Knowles (n = 120). P‐values were calculated using the Wilcoxon rank‐sum test.
**Figure S4: External validation of the LC1/LC2–3 transcriptomic classifier in the Knowles cohort (n = 120).** (**A**) Kaplan–Meier analysis of recurrence‐free survival (RFS) stratified by classifier‐predicted LC1 versus LC2/3 status. (**B**) Multivariable Cox proportional hazards analysis for recurrence, adjusting for age, sex, tumour stage, BCG treatment and presence of CIS.

## Data Availability

All data from the UROMOL cohort analysed during the current study are available as downloadable supplemental files from the original UROMOL manuscript.^19^ Data from the Knowles et al. 2021 cohort are available via Cell Reports Medicine.^24^
