# Supplementary material for: Long non‐coding RNAs define favourable biology in high‐risk non‐muscle‐invasive bladder cancer
Source: BJUI Compass. 2025 Dec 19;6(12):e70131. doi: 10.1002/bco2.70131 (PMC12715591; doi:10.1002/bco2.70131)
Supplement: Supplementary file 1 — Table S1: UROMOL 2021 classes for each of the four lncRNA clusters. Table S2: Expected frequencies for the Chi‐squared test. Table S3: Standard residuals for the X2 test comparing the lncRNA clusters with UROMOL 2021 classes. [file BCO2-6-e70131-s002.docx]

| **Table S1:** UROMOL 2021 classes for each of the four lncRNA clusters. | | | | | |
| --- | --- | --- | --- | --- | --- |
|  | *UROMOL 2021 Classification* | | | |  |
| **Cluster** | **Class 1** | **Class 2a** | **Class 2b** | **Class 3** | **Total** |
| **1** | 26 | 3 | 26 | 0 | 55 |
| **2** | 2 | 26 | 23 | 31 | 82 |
| **3** | 26 | 9 | 38 | 15 | 88 |
| **4** | 19 | 6 | 5 | 31 | 61 |
| **Total** | 73 | 44 | 92 | 77 | 286 |

| **Table S2:** Expected frequencies for the Chi-squared test. | | | | | | |  |  |  |  |  |  |
| --- | --- | --- | --- | --- | --- | --- | --- | --- | --- | --- | --- | --- |
|  | *UROMOL 2021 Classification* | | | | | |  |  |  |  |  |  |
| **Cluster** | **Class 1** | | **Class 2a** | **Class 2b** | | **Class 3** |  |  |  |  |  |  |
| **1** | 14.04 | | 8.46 | 17.69 | | 14.81 |  |  |  |  |  |  |
| **2** | 20.93 | | 12.62 | 26.38 | | 22.08 |  |  |  |  |  |  |
| **3** | 22.46 | | 13.54 | 28.31 | | 23.69 |  |  |  |  |  |  |
| **4** | 15.57 | | 9.38 | 19.62 | | 16.42 |  |  |  |  |  |  |
|  |  | |  |  | |  |  |  |  |  |  |  |
| **Table S3:**  Standard residuals for the X2 test comparing the lncRNA clusters with UROMOL 2021 classes. | | | | | | | | | | | | |
|  | | *UROMOL 2021 Classification* | | | | | | |  |  |  |  |
| **Cluster** | | **Class 1** | | | **Class 2a** | | **Class 2b** | **Class 3** |  |  |  |  |
| **1** | | 3.19 | | | -1.88 | | 1.98 | -3.85 |  |  |  |  |
| **2** | | -4.14 | | | 3.77 | | -0.66 | 1.9 |  |  |  |  |
| **3** | | 0.75 | | | -1.23 | | 1.82 | -1.79 |  |  |  |  |
| **4** | | 0.87 | | | -1.1 | | -3.3 | 3.6 |  |  |  |  |
