# Supplementary material for: Long non‐coding RNAs define favourable biology in high‐risk non‐muscle‐invasive bladder cancer
Source: BJUI Compass. 2025 Dec 19;6(12):e70131. doi: 10.1002/bco2.70131 (PMC12715591; doi:10.1002/bco2.70131)
Supplement: Supplementary file 2 — Figure S1: Consensus clustering of lncRNA expression in high‐risk NMIBC. Consensus matrix heatmap of 212 UROMOL tumours using the top 1000 variable lncRNAs. A three‐cluster solution (C1–C3) was identified, with dark blue blocks indicating stable membership across resampling iterations. Figure S2. Biological characterization of the lncRNA‐based clusters. Heatmap of the top 50 most differentially expressed protein‐coding genes across the three lncRNA‐defined clusters. Covariate tracks indicate molecular subtypes according to LundTax, TCGA, Consensus and UROMOL classifications, as well as tumour stage and patient sex. Genes were selected based on adjusted p‐value and fold‐change criteria, and highlight biological programs distinguishing each cluster. Figure S3. Biological characterization of classifier‐predicted subgroups in the Knowles cohort. Boxplots showing the distribution of signature scores across predicted LC1 and LC2/3 groups in Knowles (n = 120). P‐values were calculated using the Wilcoxon rank‐sum test. Figure S4: External validation of the LC1/LC2–3 transcriptomic classifier in the Knowles cohort (n = 120). (A) Kaplan–Meier analysis of recurrence‐free survival (RFS) stratified by classifier‐predicted LC1 versus LC2/3 status. (B) Multivariable Cox proportional hazards analysis for recurrence, adjusting for age, sex, tumour stage, BCG treatment and presence of CIS. [file BCO2-6-e70131-s001.docx]

**SUPPLEMENTAL FIGURES**

| **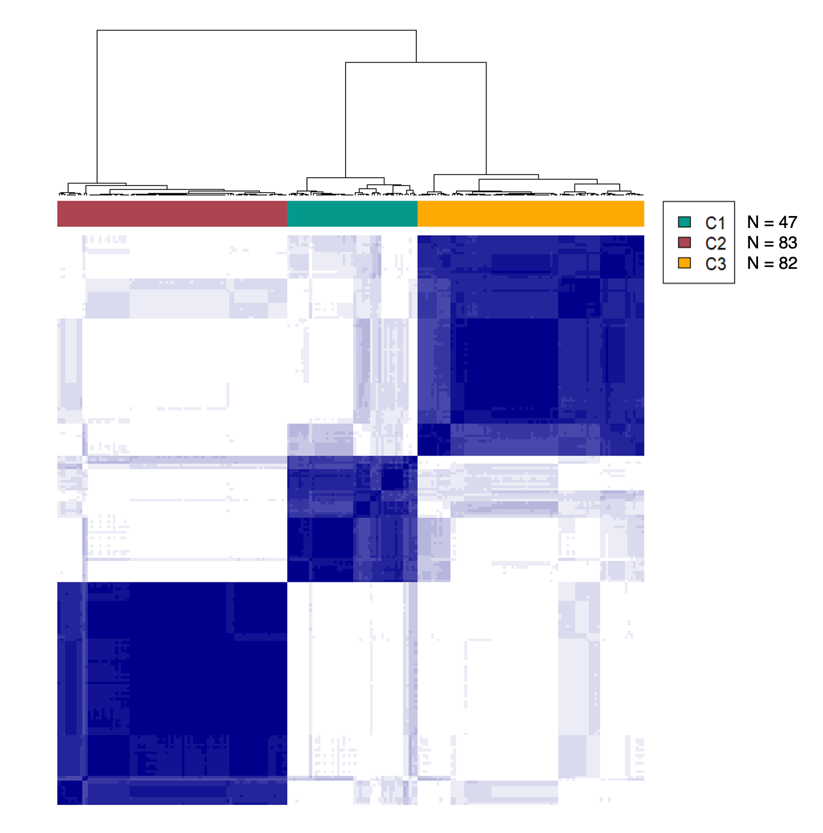** |
| --- |
| **Figure S1:** Consensus clustering of lncRNA expression in high-risk NMIBC. Consensus matrix heatmap of 212 UROMOL tumors using the top 1,000 variable lncRNAs. A three-cluster solution (C1–C3) was identified, with dark blue blocks indicating stable membership across resampling iterations. |

| 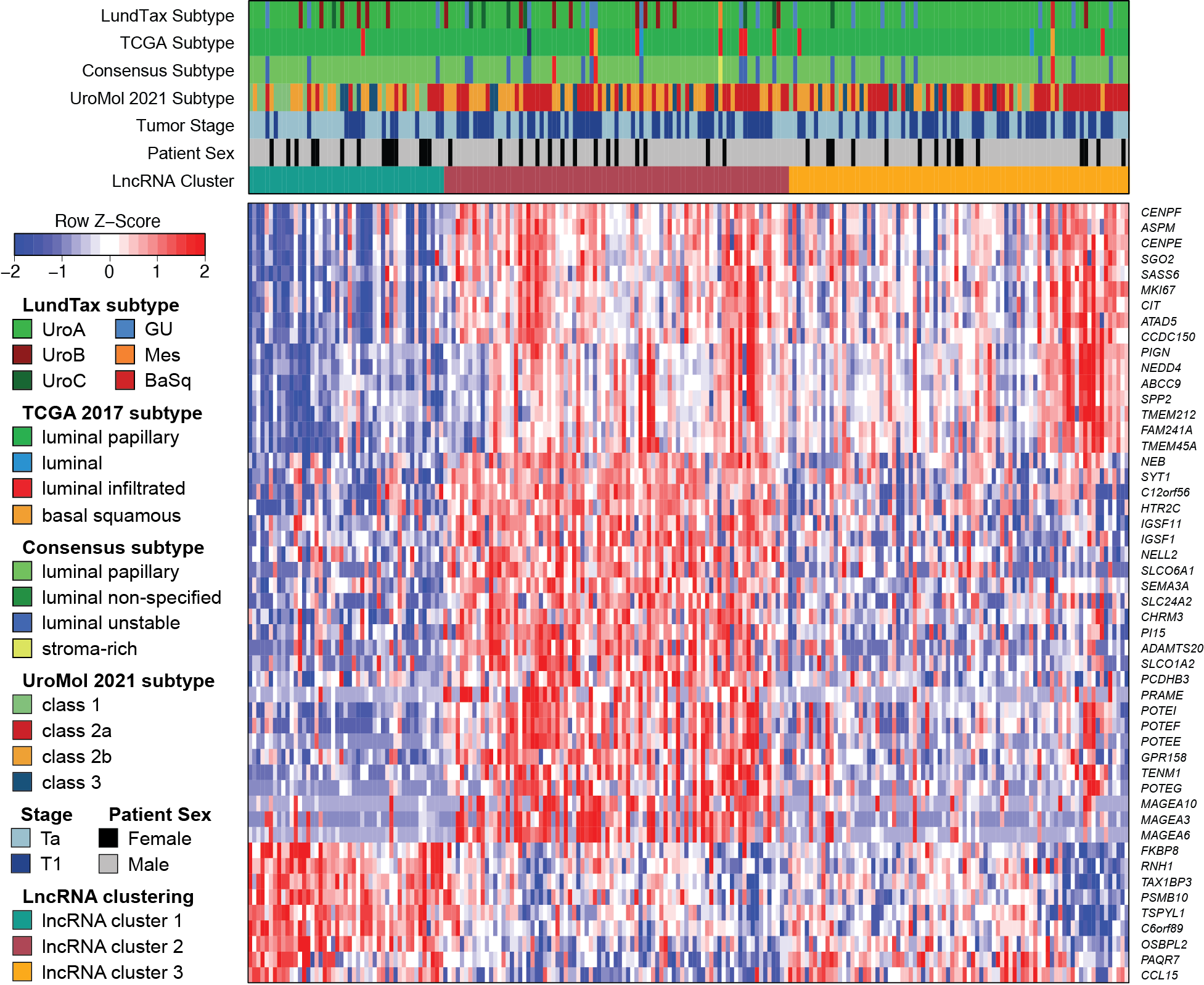 |
| --- |
| **Figure S2**. Biological characterization of the lncRNA-based clusters. Heatmap of the top 50 most differentially expressed protein-coding genes across the three lncRNA-defined clusters. Covariate tracks indicate molecular subtypes according to LundTax, TCGA, Consensus, and UROMOL classifications, as well as tumor stage and patient sex. Genes were selected based on adjusted p-value and fold-change criteria and highlight biological programs distinguishing each cluster. |

| **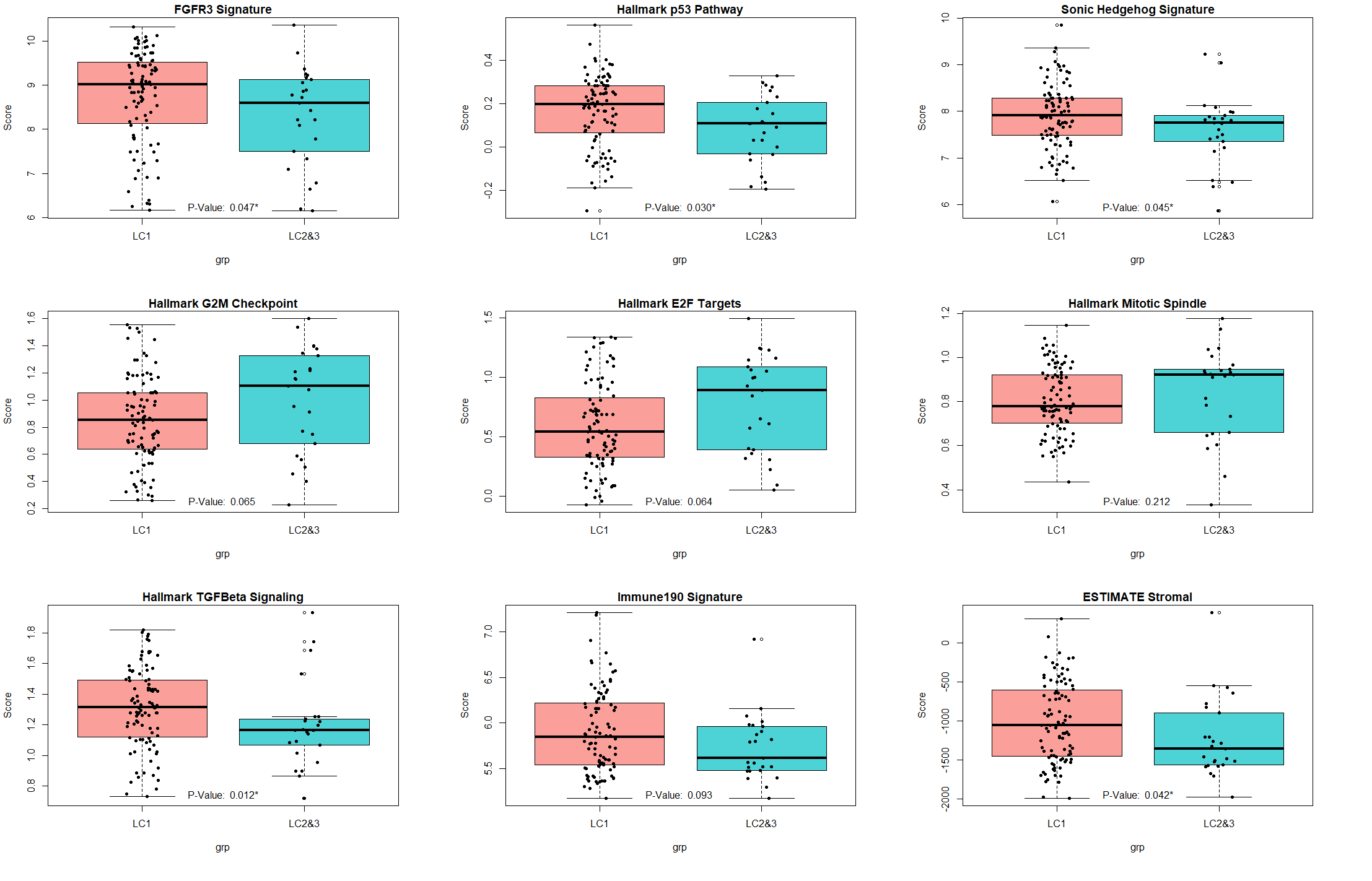** |
| --- |
| **Figure S3. Biological characterization of classifier-predicted subgroups in the Knowles cohort.** Boxplots showing the distribution of signature scores across predicted LC1 and LC2/3 groups in Knowles (n = 120). P-values were calculated using the Wilcoxon rank-sum test. |

| **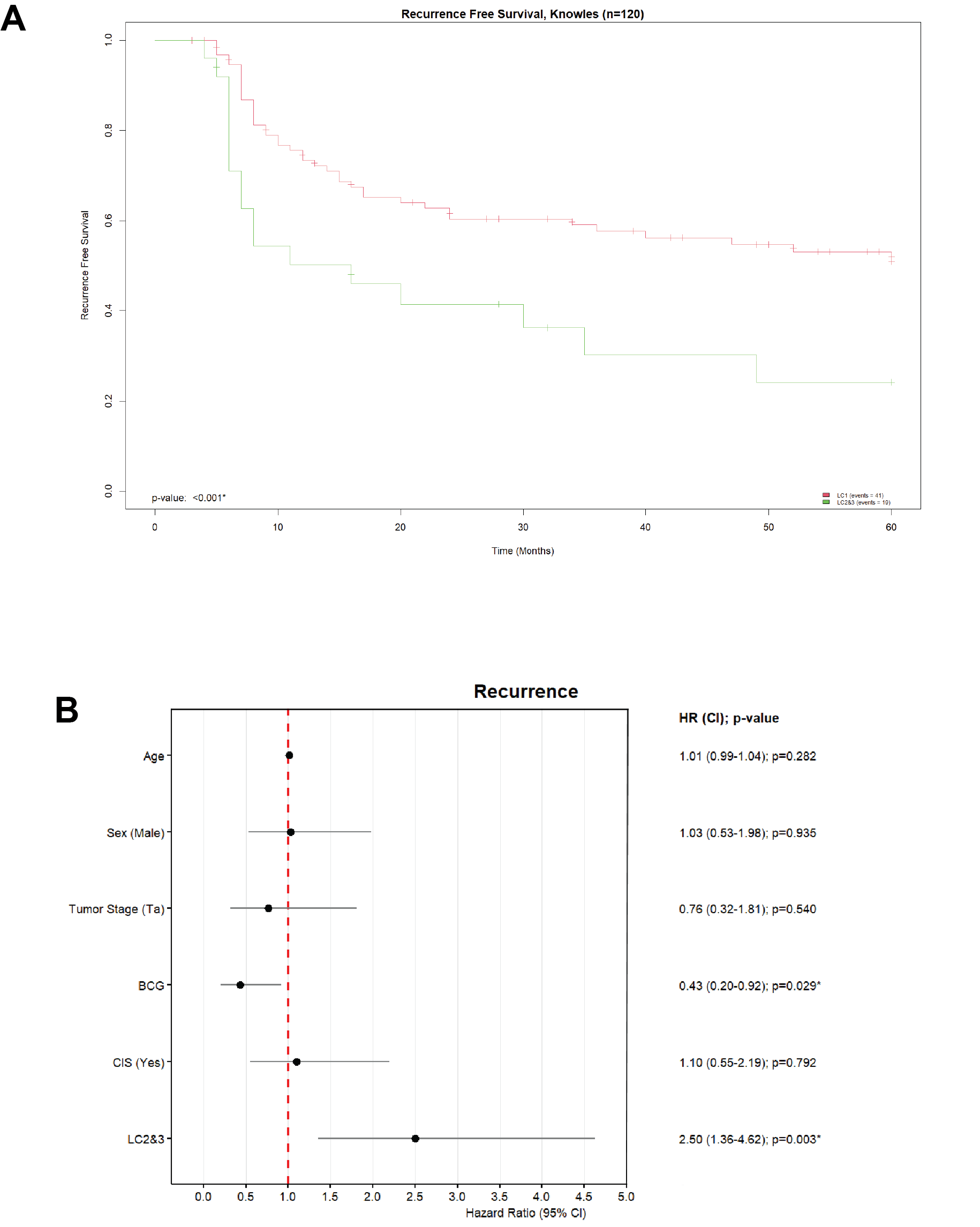** |
| --- |
| **Figure S4: External validation of the LC1/LC2-3 transcriptomic classifier in the Knowles cohort (n = 120).** (**A**) Kaplan–Meier analysis of recurrence-free survival (RFS) stratified by classifier-predicted LC1 versus LC2/3 status. (**B**) Multivariable Cox proportional hazards analysis for recurrence, adjusting for age, sex, tumor stage, BCG treatment, and presence of CIS. |
